# The impact of avoiding cardiopulmonary by-pass during coronary artery bypass surgery in elderly patients: the Danish On-pump Off-pump Randomisation Study (DOORS)

**DOI:** 10.1186/1745-6215-10-47

**Published:** 2009-07-04

**Authors:** Kim Houlind, Bo Juul Kjeldsen, Susanne Nørgaard Madsen, Bodil Steen Rasmussen, Susanne Juel Holme, Thomas Andersen Schmidt, Poul Erik Haahr, Poul Erik Mortensen

**Affiliations:** 1 Department of Cardiothoracic and Vascular Surgery, Aarhus University Hospital, Skejby, Denmark; 2 Department of Cardiothoracic Surgery, Odense University Hospital, Odense, Denmark; 3 Department of Cardiothoracic Surgery, Center for Cardiovascular Research, Aalborg Hospital, Aarhus University Hospital, Aalborg Denmark; 4 Department of Anaesthesia, Aalborg Hospital, Aarhus University Hospital, Aalborg, Denmark; 5 Department of Cardiothoracic Surgery, Gentofte Hospital, Hellerup, Denmark

## Abstract

**Background:**

Coronary Artery Bypass Graft operation for ischemic heart disease provides improved quality of life and, in some patients, prolonged survival. Concern has, however, been raised about complications that may be related to the use of cardiopulmonary by-pass (CPB) and aortic cross-clamping. It has been hypothesized that when coronary artery by-pass grafting is performed without the use of CPB, the rate of serious complications is reduced.

**Methods/Design:**

The trial is designed as an open, randomized, controlled, clinical trial with blinded assessment of end-points. Patients at or above 70 years of age, referred for surgical myocardial revascularisation, are included and randomised to receive coronary artery by-pass grafting either with or without the use of CPB and aortic cross-clamping. Follow-up is performed by clinical, biochemical, electrocardiographic, and angiographic data that are evaluated by independent committees that are blinded with respect to the result of the randomisation. End points include mortality, stroke, myocardial infarction, graft patency, quality of life, and cost-effectiveness. The trial is performed in four different Danish, cardiac surgery centres.

**Trial registration:**

ClinicalTrials.gov NCT00123981

## Background

Conventional Coronary Artery By-pass Grafting (CCABG) using CPB has for decades been applied to obtain myocardial re-vascularisation and, hence, improved quality of life and survival. It does, however, bear a risk of death, stroke, myocardial infarction and other serious complications.

During recent years, an equivalent operation performed on the beating heart without cardiopulmonary bypass (Off-pump Coronary Artery By-pass Grafting, OPCAB) has gained popularity helped by the advent of mechanical stabilization devices and improved surgical techniques. Observational studies suggest that this technique is associated with a lower incidence of stroke, per operative arrhythmias and even mortality than conventional CCABG [1]. This is especially the case in elderly patients and patients with significant co-morbidity [2-4].

Only few randomised, controlled trials have been conducted [5-9] and most of these included mainly or only low-risk, relatively young patients. These studies have documented the safety and efficacy of OPCAB compared with CCABG, but none of the trials has had the statistical power to determine whether the rate of serious complications is lower after OPCAB operations. In contrast to earlier findings [5], one recent study found graft patency to be significantly lower after OPCAB than after CCABG operations [9].

We find that there is a need of a larger scale randomised trial to compare the results of CCABG and OPCAB operations, especially in elderly patients.

### Aims

Primarily, to compare the incidence of death, stroke and myocardial infarction after CCABG and OPCAB procedures in a population of elderly patients. Secondly, to compare quality of life and graft patency, and cost-effectiveness after CCABG and OPCAB.

## Design

An open, randomised Danish multicentre trial of patients 70 years of age or above, admitted for surgical, first time myocardial re-vascularisation. The design involves blinded evaluation of outcome measures and interim analyses.

### Participating centres

The study will be performed at the cardiac surgery units of Aalborg Hospital, Aarhus University Hospital, Gentofte Hospital, Odense University Hospital, and Aarhus University Hospital, Skejby, Denmark.

### Approval to start

Patients will be enrolled in the study based upon written informed consent in accordance with Helsinki Declaration II. The study plan has been approved by the local ethical committees and the national ethical committee.

### Trial surgeon's requirements

Before being certified to participate, the surgeons must at least independently have performed 25 CCABG and 25 OPCAB procedures each procedure including anastomoses to the marginal tbranches of the left coronary artery. This territory is the most challenging to graft. Therefore, all surgeons fulfilling this criterium will first have performed a significant number of other coronary artery by-pass operations and have an intermediate level of experience reflecting common clinical situations. Based upon these criteria and on documented results of the operations, surgeons must also be certified by the ethical and safety data committee.

### Inclusion criteria

Patients age seventy years or above admitted for first time coronary artery by-pass operation without other planned surgical procedure.

### Exclusion criteria

- Patient not willing to participate in the study

- The patient cannot understand given information or answer questionnaires relevantly due to intellectual or linguistic deficiency

- Preoperative knowledge that aortic cross clamping is not safe due to calcification e.g. from CT-scan.

- Preoperative cardiac conditions demanding CPB.

- Re-do cardiac surgery.

- Acute operation defined as patient requiring operation before the beginning of the next working day after first being presented to the surgeon.

- Any other reason why the operating surgeon does not believe that the patient can be operated safely either using CPB or without CPB.

- Inclusion in study not possible for logistic reasons

### Consent and inclusion procedure

After written and oral information is given by the project nurse at the ward at time of admission for coronary artery by-pass grafting, the patient has to give oral and written consent.

### Randomisation

The operating surgeon enters patients in a central database accessed by the internet. Before being able to randomise, the surgeon has to enter a preoperative plan including which coronary arteries are to be grafted, the conduit type and whether a single, sequential, or y-graft is planned. Having done this the patient is subsequently randomised either to OPCAB or CCABG by the on-line system

### Blinding

For practical end ethical reasons, neither the patient nor doctors or nurses are blinded with respect to the type of treatment used. Events will, however, be evaluated by an independent committee that will be blinded.

### Sample size

900 patients including 450 patients randomised for OPCAB and 450 randomized for CCABG.

### Power calculations

The incidence of the primary, combined end-point is estimated to be 8%. This estimate is based on literature as well as on historical data from the participating centres from the period 2001–2003. Based on earlier, observational studies of elderly patients undergoing coronary revascularisation [2,3], we hypothesize that this incidence can be reduced to 4% by avoiding cardiopulmonary by-pass. With a margin of 0.5% in risk difference a test of non-inferiority of OPCAB compared to CCABG based on 900 patients will have a statistical power of 82%.

### Operations and anaesthesia

The coronary artery by-pass operations are performed either with or without the use of CPB according to the result of randomisation. Operative protocols are used to limit the variations of technique, although leaving some room for variations according to the discretion of the individual surgeon and the tradition of each participating unit. The operative protocols are given in Appendix 1. When judged vital for the outcome of the patient, conversion from one group to the other can be decided by the operating surgeon at any time during the operation. Equally, anaesthetic protocols are followed in order to limit the variations between the units (Appendix 2)

### Postoperative treatment

Postoperative treatment of included patients with acetyl salicylic acid, clopidogrel, angiotensin converting enzyme inhibitors, diuretics, amiodarone and heparin will be performed according to detailed protocols (Appendix 3)

In case deviation from the protocol is deemed necessary, such altered strategies are to be duly noted and accounted for.

### Follow-up

*At preoperative conference: *Evaluation of whether the patient meets the inclusion and/or exclusion criteria. All patients above 70 years who are to have a sole by-pass operation are registered. If the patient is not included in the project, the reason is noted.

*Day one: *The day the patient is admitted to the hospital for operation. Informed oral and written information and consent. Questionnaires are answered.

*Day two: *Randomization. CCABG or OPCAB are performed according to randomization.

*Day 31: *Telephone contact and ECG. Evaluation of primary end-point.

*Six months postoperatively: *Coronary angiography. Clinical control and questionnaire.

*Three years postoperatively: *Clinical control and questionnaire.

During follow-up the patients will be followed up through unambiguous record linkage with population-based registries by using the civil registry number, which is a unique identification code given to every person residing in Denmark at birth or upon immigration. Information on death will be obtained from the Civil Registration System, which keeps electronic records on all changes in vital status, including change of address, date of emigration, and date of death for the entire Danish population.

Information on hospitalizations for myocardial infarction and stroke will be obtained from The National Registry of Patients, which retains key information on all patients discharged from non-psychiatric hospitals in Denmark since 1977, including outpatient diagnoses, and patients dying during their admission. The files of the registry include information on the civil registry number of the patient, date of discharge, and up to 20 discharge diagnoses, assigned exclusively by the physician at discharge according to the Danish version of the International Classification of Diseases, 10th revision (ICD-10) thereafter [10]. Full medical records including results of paraclinical examinations and tests will be retrieved and reviewed according to standardized internationally accepted criteria for all patients who die and/or are registered with a discharge diagnosis of acute myocardial infarction or stroke during the study period. All data will be presented for the independent monitoring committee.

### Primary endpoint

A combined end-point of death + stoke + myocardial infarction within 30 days postoperatively. Definitions of end-points will be according to appendix 4.

### Secondary endpoints

a) A combined end-point of death + stoke + myocardial infarction during follow-up

b) Patency of by-pass grafts assessed by coronary angiography 6 months after the operation.

c) Total mortality and cardiac mortality during follow-up.

d) Need of new intervention for cardiac angina during follow-up.

e) Quality of life assessed by MOS SF-36 and EuroQol questionnaires 6 months and 3 years after the operation.

f) Total hospital costs and costs of public care provided 6 months and 3 years after the operation and difference in costs per quality adjusted life year.

### Analysis

Kaplan-Meier plots will be used for graphic comparisons between groups and log- rank test for numerical comparison. Data analysis will be performed in accordance with the principle of intention-to-treat. An interim analysis will be performed for monitoring the accumulating data during the planned study period, i.e., after half of the patients have been recruited. The O'Brien-Fleming approach will be used in these analyses. At the interim analysis a p-value below 0.5% will be considered significant, while a p value below 4.8% will be considered significant after conclusion of the study. In this manner, a 5% level of significance is sustained. For the 6 months and 3 years data, Kaplan-Meier plots will be used for graphic comparisons between groups and log- rank test for numerical comparison.

## Competing interests

The authors declare that they have no competing interests.

## Authors' contributions

All authors were involved in the conception and design of the study protocol, drafting or revising of the manuscript, and have approved the final manuscript.

## Appendices

### Appendix 1

#### A Standard off-pump (OPCAB) procedure

1. Full median sternotomy is used in all patients

2. Graft material is harvested and controlled

3. Heparin is given and the Activated Clotting time (ACT) is controlled. If necessary, additional heparin is given to achieve an ACT above 400 sec. before arteriotomy.

4. Autotransfusion or cellsaver is used to avoid blood spill.

5. Opening of the pericardium

6. Devices for mobilization and stabilizing the heart are positioned

7. The vessel is opened

8. An intracoronary shunt is placed and/or CO- blower may be used. If necessary, a snare can be placed proximally but not distally to the anastomosis

9. The anastomosis is made with prolene 7.0 running suture

10. The proximal anastomoses are performed either before or after performing the distal anastomosis with minimal manipulation of the aorta. Bilateral internal mammary artery grafts, Y-grafts can be used but also proximal anastomosis devices and side-clamping are allowed.

11. Flow in coronary grafts is controlled using transit-time flow meter and registered. Eventual redo of the anastomosis is at the surgeon's discretion.

12. Two atrial and two ventricular pace electrodes are placed.

13. After the procedure, 4 mg of protamin is given and if necessary additional protamin in order to fully revert the heparinization to ACT level below 120 s.

14. Drainage tubes are placed.

i) Sternum is closed with wires and fascia, subcutis and skin closed with sutures.

#### B Standard CCABG procedure

1. Full median sternotomy is used in all patients

2. Graft material is harvested and controlled

3. Heparin is given and the Activated Clotting time (ACT) is controlled. If necessary, additional heparin is given to achieve an ACT above 400 sec. before arteriotomy.

4. The pericardium is opened and pericardial sutures are placed to expose the heart.

5. Cannulation of right atrium and aorta. Start of cardiopulmonary by-pass.

6. When the heart is fully relieve, the places where to place the anastomoses is marked.

7. Cross-clamping of the aorta

8. Cold antegrade or retrograde cardioplegic solution is given. Both crystalloid and hemocardioplegia are accepted

9. Graft material is prepared for anastomosis when asystoli has occurred and the ordered amount of cardioplegic solution is given, distal anastomoses are made using 7.0. prolene running suture.

10. Patency and tightness of the anastomosis are tested using cardioplegic solution if distal anastomoses are performed before proximal anastomoses.

11. The proximal anastomoses are performed either before or after performing the distal anastomosis. Bilateral internal mammary artery grafts, Y-grafts can be used but also proximal anastomosis devices and side-clamping are allowed.

12. Flow in coronary grafts is controlled using MediStim Transit-time flow meter and registered. Eventual redo of the anastomosis is at the surgeon's discretion.

13. Extra-corporeal circulation is gradually decreased and stopped. Test-dose of protamin is given.

14. Decannulation.

15. After the procedure, protamin is given to fully revert the heparinization to ACT level below 120 s.

16. Two atrial and two ventricular pace electrodes are placed.

17. Drainage tubes are placed.

18. Sternum is closed with wires and fascia, subcutis and skin closed with sutures.

### Appendix 2. Anaesthesia

The aim is that all patients receive a standard anesthesia and perioperative treatment, to minimize the influence of anesthesia and perioperative care in the included patients.

The following is recommendated guidelines. There will be slight differences between the involved hospitals, but it is mandatory for each hospital to secure, that there will be no differences is anesthesia and perioperative care between the two groups (OPCAB and CCABG).

#### Premedication

Peroral midazolam 3.75–7.5 mg one hour prior to surgery. If the patient receives treatment with beta-blocker, the usual dose is given at the same time.

#### Anesthesia

Intravenous induction with midazolam (0.05–0.15 mg/kg) and fentanyl (5–15 μg/kg); muscle relaxation with rocuronium (0.6 mg/kg). Anesthesia shall be maintained with either inhalation of sevoflurane or desflurane to improve ischemic preconditioning and with additional boluses of fentanyl (total dose, 20–30 μg/kg) and rocuronium. During cardiopulmonary bypass (CPB) the inhalation agent is given to the bypass circuit (Aarhus University Hospital, Aalborg and Odense University Hospital), alternatively an infusion of propofol is given to the bypass circuit (Aarhus University Hospital, Skejby and Gentofte Hospital). Aarhus University Hospital, Skejby will use total intravenous anesthesia due to lack of equipment for inhalation agent.

#### Monitoring

Arterial catheter; central venous catheter; pulmonary artery catheter for measuring of central venous pressure, pulmonary artery pressure and cardiac output, preferable a fiberoptic catheter shall be inserted for measuring of mixed venous saturation (S_V_O_2_), 5-lead ECG for measuring of heart rate and cardiac ischemia, peripheral oxygen saturation (S_p_O_2_), end-tidal carbon dioxide (CO_2_); Minimal alveolar concentration (MAC) of anesthetic inhalation agent; central body temperature (bladder); peripheral temperature; catheter in the bladder.

All parameters will be recorded continuously according to the flow chart in each department. Specific defined parameters will be recorded in the flow chart following the present study.

#### Temperature

Normothermia is defined as a central body temperature (bladder) of 35–37°C.

CABG group: The central body temperature shall be achieved by a heat-exchanger in the cardiopulmonary bypass circuit.

OPCAB group: The central body temperature shall be achieved by using warm intravenous fluids, a heating mattress, a warming blanket, in addition to maintaining a warm operation theatre.

#### Ventilation

Mandatory ventilation tidal volume 8–10 ml/kg and the rate adapted for a partial arterial pressure of CO_2 _(PaCO_2_) between 4.5 and 5.5 kPa, positive end-expiratory pressure (PEEP) at 5 cmH_2_O, and FiO_2 _at 0.4–0.6 to keep partial arterial pressure of oxygen (PaO_2_) above10 kPa. Patients with preoperative chronic obstructive lung disease shall at least be treated in accordance to their preoperative values of PCO_2 _and PO_2_.

Before closure of sternum, the lungs shall be inflated manually to reopen atelectasis due to pulmonary collapse during surgery.

Extubation as soon as the drainage loss in acceptable; the central body temperature is >36°C and peripheral temperature is >32°C; the circulation is stabilized with a mean arterial pressure > 70 mmHg, a cardiac index > 2.2 liters/minut/m^2 ^and a heart rate < 90/minute, and if measured a SvO_2_>60%; normocapnia and oxygenationindex (PaO_2_/FiO2) > 35.

After extubation in intensive care unit (ICU) the aim is a PO_2_>10 kPa achieved by supplementary treatment with either dry oxygen 1–5 litres/minute or humidified oxygen 15 litres/minute with FiO_2 _between 0.40 and 1.0.

#### Vasoactive drugs

Vasodilators: Nitroglycerine, nitroprusside, nifidipine.

Vasocontractors: Noradrenaline.

Inotropic agents: Epinephrine, dobutamine, dopamine, phosphodiesterase inhibitors, calcium sensitizer.

The vasoactive drugs are given on indication to secure optimal central and peripheral circulation. The drug of choice depends of the routine in the cardiac units.

#### Mechanical support

Intraaortic ballon pump and assist device is placed on indication depending on the routine in the cardiac units.

#### Volume substitution

Crystalloids: Isotonic saline, isotonic ringers acetate, isotonic potassium-glucose.

Colloids: Hydroxyethyl starch, dextran.

Blood products: On indication according to the recommendations on the centres.

Fluids are given to maintain normovolemia and hematocrite > 0.24.

#### Blood glucose

All patients, non-insulin dependent and insulin dependent diabetes as well as patients without diabetes shall have blood glucose monitored carefully during surgery and in ICU.

The goal of blood glucose level shall be:

Peroperative blood glucose: 6 – 10 mmol/l

Postoperative (ICU) blood glucose: 5 – 7 mmol/l

The maintenance of blood glucose level shall be achieved by infusion of glucose-insulin-potassium according to the regime in each hospital.

#### Postoperative sedation

Infusion of propofol 0.5–2 mg/kg/hour and incremental dosis of morphine 1–4 mg. Supplementary NSAID is given according to the regimes in the cardiac units.

#### Antiemetics

Bolus dose of metoclopramid or ondansetron on indication.

### Appendix 3. Postoperative treatment

Low molecular heparin: Fragmin 5000 IE × 1 untill 7th postoperative day or discharge from hospital

Acetyl-salicylic-acid: 75 mg × 1

#### Clopidogrel (Plavix)

75 mg × 1 for three months. Patients who have started clopidogrel treatment because of earlier implanted stent or because of acute myocardial infarction will continue the planned treatment. These patients shall also receive clopidogrel for at least 3 months postoperatively.

#### Statines

All patients shall receive treatment with statins. Thyroid and liver biochemical analyses are controlled before beginning treatment. In case of planned operations, treatment is paused at the day of admission to hospital but is started again before discharge

#### Acute operations

treatment is paused at the day of the operation but is started again before discharge.

#### Dose

Pravachol 40 mg × 1, Zocor 20–40 mg × 1, Simvastatin 40 mg × 1 or Zarator 10 mg × 1.

#### ACE-inhibitors

Shall be used in patients with left ventricular ejection fraction < 50%, congestive heart failure and/or arterial hypertension.

#### Betablockers

In patients with acute myocardial infarction within the last year and/or congestive heart failure.

#### Diuretics and potassium

Congestive heart failure with fluid retention and in some patients with arterial hypertension.

#### Amiodarone

In patients clinically affected by postoperative atrial fibrillation, initially a bolus of 300 mg is injected intravenously within 20 minutes, followed by infusion of 1200 mg the following 24 hours or untill conversion to sinus rhythm. Infusion through central venous catheter. Bolus injection can, however be performed through peripheral vein.

Subsequently, oral treatment is T. Cordarone 600 mg × 2 for one week, 400 mg × 2 second week, 200 mg × 2 third week and 200 mg × 1 during the fourth week followed by discontiuation of therapy in case of stable sinus rhythm.

### Appendix 4. Definitions

#### A. Postoperative acute myocardial infarction

1. Postoperative acute myocardial infarction: A peak creatine kinase-MB(CK-MB) of at least 75 ng/ml by day 3

2. New Q-wave evidence of MI, along with CK-MB of at least 50 ng/ml by day 3

3. New Q-wave evidence of MI by day 30 that was not present at day 3

4. MI (Q-wave or non-Q-wave) as defined by the World Health Organisation

#### B. Stroke

The World Health Organization's definition of stroke is used, i.e., an acute disturbance of focal or global cerebral function with symptoms lasting more than 24 hr or leading to death of presumed vascular origin.

Patients with the following ICD-10 discharge diagnosis codes in the Danish National Patient Registry are considered possible cases of stroke:

I 61 Intracerebral haemorrhage

I 63 Ischaemic stroke

I 64 Non-specified stroke

Patients with subdural haematoma, epidural bleeding, retina infarction or infarction caused by subarachnoidal bleeding, trauma, infection, surgery (not related to CABG or OPCAB surgery) or a intracerebral tumor are not classified as stroke patients. Moreover the definition does not include patients with only non-specific symptom, e.g. isolated dizziness or headache or asymptomatic patients with infarctions identified only by brain imaging.

Diagnostic work-up: In case of clinical symptoms of stroke or transient cerebral ischemia (TCI), symptoms should be noted in the patient file and the neurologist on duty should be asked to make a formal examination of the patient. All possible cases of stroke or TCI should undergo a brain imaging procedure, preferably a MR-diffusion scan. Should a MR-diffusion scan not be possible, efforts should be made for the patient to have a CT scan 3–7 days after the start of symptoms (even if a CT scan has been made in the acute phase).

#### C. Graft patency

The coronary angiogram is evaluated by two different cardiologists. The grafts are rated A: Open, B: Stenotic (more than 50%), O: Occluded, each for the 1) distal anastomosis, 2) proximal anastomosis og 3) length of the graft. Discrepancies are settled by conference.
